# The importance of skin prick test in differentiating cat sensitivities and allergies in children

**DOI:** 10.55730/1300-0144.5650

**Published:** 2023-05-31

**Authors:** Mehmet ŞİRİN KAYA, Özge ATAY, İdil AKAY HACI, İlke TAŞKIRDI, Selime ÖZEN BÖLÜK, Figen ÇELEBİ ÇELİK, Özgen SOYÖZ, Canan Şule KARKINER, Demet CAN

**Affiliations:** 1 Department of Pediatric Immunology and Allergy, Dr. Behçet Uz Child Disease and Pediatric Surgery Training and Research Hospital, İzmir, Turkiye; 2 Department of Pediatric Immunology and Allergy, İzmir University of Health Sciences, Tepecik Training and Research Hospital, İzmir, Turkiye; 3 Department of Pediatric Immunology and Allergy, Aydın Gynecology and Pediatrics Hospital, Aydın, Turkiye

**Keywords:** Allergy, cats, child, diagnosis, prick test

## Abstract

**Background/aim:**

The incidence of cat allergies in children has increased over the years. Children with cat allergies have mostly reported respiratory symptoms. The skin prick test (SPT) is the most preferred method to demonstrate sensitization to allergens. However, not all children who develop cat sensitization due to environmental exposure become allergic to cats. In our study, we aimed to determine the frequency of sensitization to cat and cat allergy, cat-related symptoms, and the cut-off value for the SPT that may indicate cat allergy.

**Materials and methods:**

Patients aged 2–18 years, who applied to the Health Sciences University İzmir Dr Behçet Uz Pediatrics and Surgery Training and Research Hospital and Balıkesir University Application and Research Hospital Pediatric Allergy outpatient clinics between January 01, 2019 and December 31, 2020, were included in the study. Patients who underwent SPT and found to be sensitized to cat allergen, were evaluated retrospectively. Clinical and laboratory findings of the patients were recorded. Receiver operating characteristics (ROC) analysis was performed to determine the cut-off value for the SPT.

**Results:**

Sensitization to cat was detected in 140 (4%) out of 3499 patients who underwent SPT. The median age of the patients was 12 years (min–max: 5–18) and 67.1% were male. Eighty-eight (62.9%) patients were symptomatic upon contact with cats, predominantly with nasal symptoms. These patients had significantly larger cat SPT wheal size than asymptomatic patients. The cut-off value was determined as 5.5 mm with a sensitivity of 72.7% and a specificity of 61.5% (95% CI, 60.5%–78.4%). Symptoms resolved in about half of our patients by reducing contact with cats.

**Conclusion:**

The present study is the first to report the frequency and clinical findings of cat sensitizations and allergies in Turkish children. For effective treatment, cat allergy must be diagnosed. In this regard, the use of a practical, readily accessible 5.5 mm cut-off point on the SPT may be helpful.

## 1. Introduction

Cats have been living with humans for centuries and are one of the most popular pets. An increasing incidence of allergy to cats has been reported over the years. The symptoms of cat allergy range from mild rhinoconjunctivitis to potentially life-threatening asthma exacerbations and anaphylaxis [1–3].

To exclude or confirm a suspected IgE-mediated allergy to animals, it is recommended to use skin prick test (SPT) with standardized extracts in addition to taking medical history and physical examination [4]. Although specific exposure tests are the gold standard test for assessing the clinical significance of sensitization to an allergen, there is insufficient evidence for the practical use and standardization of these tests in feline allergy [5].

People who keep cats at home are more likely to develop sensitization. However, it has been reported that cat allergens adhere to the clothes of people living at home, causing them to be transported to various environments shared by the community, and sensitization has been reported to increase in these people [6,7].

In addition, an increased risk of sensitization to other animal allergens, especially to dogs, has been reported in cat-sensitized patients [5,8].

The most effective ways recommended to reduce symptoms in people with cat allergy are removing the pet from the house, reducing contact, and administering allergen-specific immunotherapy to eligible patients [9].

In our study, we aimed to evaluate the frequency of cat sensitization, other allergen sensitizations accompanying cat sensitization, rates of cat petting at home, presence of cat-related symptoms, and to determine the cut-off value for the skin prick test (SPT) that may indicate cat allergy. In addition, we evaluated the precautions taken by patients with feline sensitization.

## 2. Materials and methods

### 2.1. Population

This retrospective cross-sectional study was conducted in the Pediatric Allergy outpatient clinics of University of Health Sciences, İzmir Dr. Behçet Uz Pediatrics and Surgery Training and Research Hospital and Balıkesir University Application and Research Hospital between January 01, 2019 and December 31, 2020.

Patients aged between 2–18 years with allergic symptoms were evaluated. Those who underwent skin prick testing and found to be sensitized to cat allergen were included in the study.

The patients who applied to our clinic were routinely questioned if they have had any animals and/or the presence of allergic reactions related to animal contact and the findings were recorded. Patient charts and outpatient clinic files of patients with sensitization to cats were retrospectively analyzed. We examined the cat feeding status of the patients and the presence of allergic symptoms in contact with cats. Demographic data and laboratory findings (Total IgE and absolute eosinophil counts) were recorded. Patients reporting allergic symptoms such as eye symptoms (watering, redness, and itching), nasal symptoms (sneezing, itchy nose, runny nose, and nasal congestion), skin changes (atopic dermatitis, urticaria, erythema) and respiratory symptoms (cough, shortness of breath, and wheezing), after exposure to cat, were enrolled. Patients were divided into two groups according to their symptom development upon contact with a cat. Those who developed at least one allergy symptom were considered symptomatic and those who did not were considered asymptomatic.

The presence of bronchial asthma, atopic dermatitis, and allergic rhinitis were investigated by an allergist. The diagnosis of asthma was defined according to the Global Initiative for Asthma guidelines, the diagnosis of atopic dermatitis was defined according to the revised Hanifin and Rajka criteria, and the diagnosis of allergic rhinitis (AR) was defined according to the ARIA guidelines [10–12].

We recorded whether the patients owned a cat, the measures taken to reduce symptoms, and the effects of these measures.

### 2.2. Skin prick test

Skin tests were completed with similar techniques and tools, avoiding the use of conditions and drugs that would adversely affect the skin tests. Pollen (Grasses, Artemisia vulgaris, Betula alba, and Olea europaea), house dust mites (Dermatophagoides pteronysinua and farinae), animal dander (Felis domesticus, Canis familiaris, and Blatella germanica), and mold (Alternaria alternata) allergens were used in the SPT (Alk-Abello^®^, Hørsholm, Denmark). SPT was considered positive if the wheal size of any allergen was ≥3 mm relative to the negative control. If sensitization to a different allergen was detected in addition to sensitization to cat allergen, the individual was considered to be multisensitized.

### 2.3. Ethics committee

The approval of the Clinical Research Ethics Committee of İzmir Dr. Behçet Uz Pediatrics and Pediatric Surgery and Research Hospital was obtained (approval number: 2021/578).

### 2.4. Statistical analysis

The Kolmogorov–Smirnov normality test was performed to determine the statistical methods to be used. Nonparametric test methods were used if any of the groups did not meet the normality assumption. Within this scope, the Mann-Whitney U test was used to compare the variables obtained by measurement in two independent groups, while the chi-square and Fisher exact tests were used to analyze the relationships among categorical variables or intergroup differences. Receiver operating characteristics (ROC) analysis and Youden J criteria based on the sum of the highest sensitivity and specificity for a diagnostic test were used to determine the optimum cut-off value for the SPT wheal size indicating cat allergy. Statistical analysis of the study was performed using IBM SPSS Statistics version 25 for Windows, and the level of statistical significance was set at p ≤ 0.05.

## 3. Results

Cat sensitization was detected in 140 (4%) of 3499 patients who applied to our clinic with any allergic symptoms and underwent SPT between January 01, 2019 and December 31, 2020. None of them had restricted cat contact before SPT.

The median age was 12 years in 140 patients with sensitization to cat (min–max: 5–18) years and 67.1% were male. Allergic symptoms were reported by 88 (62.9%) patients upon contact with cats. The most common symptoms were related to allergic rhinitis; 51.4% of patients had sneezing, 32.1% had runny nose, and 26.4% had nasal itching. Shortness of breath (24.3%), cough (16.4%), rash (15.7%), and wheezing (9.8%) were the other symptoms described by the patients.

**Figure 1:**
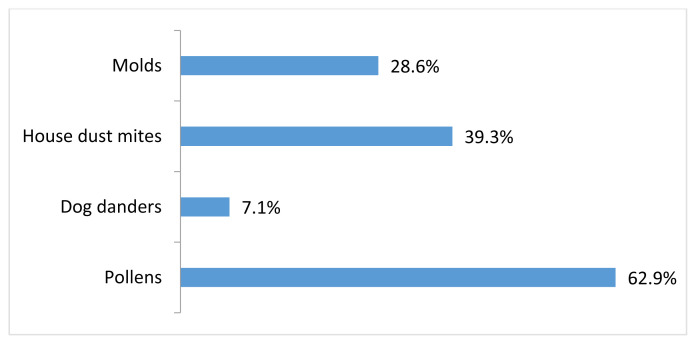
Other aeroallergens accompanying cat sensitization.

According to the results of the SPT, multisensitivity was detected in 119 (85%) patients. Sensitization to cats was most commonly accompanied by sensitization to pollens, especially olive pollens (Figure 1). There was no significant difference between single cat-sensitive and multisensitive patient groups in terms of the presence of nasal itching, runny nose, sneezing, shortness of breath, cough, and rash (p = 0.436, p = 0.526, p = 0.297, p = 0.246, p = 0.774, p = 0.649, respectively).

Demographic, clinical and laboratory findings of the patients are summarized in Table 1. There were 38 (27.1%) patients who had cats at home and 34 of these patients were symptomatic. The remaining 54 symptomatic patients did not have cats in their home. Symptomatic patients were significantly more likely to have cats at home than asymptomatic patients (p < 0.001). Total IgE, AEC (absolute eosinophil count), and SPT wheal size for cat allergen were significantly higher in symptomatic patients compared to asymptomatic patients. Similarly, these parameters were found to be higher in those who petted cats at home than those who did not (p < 0.001) (Table 2).

**Figure 2:**
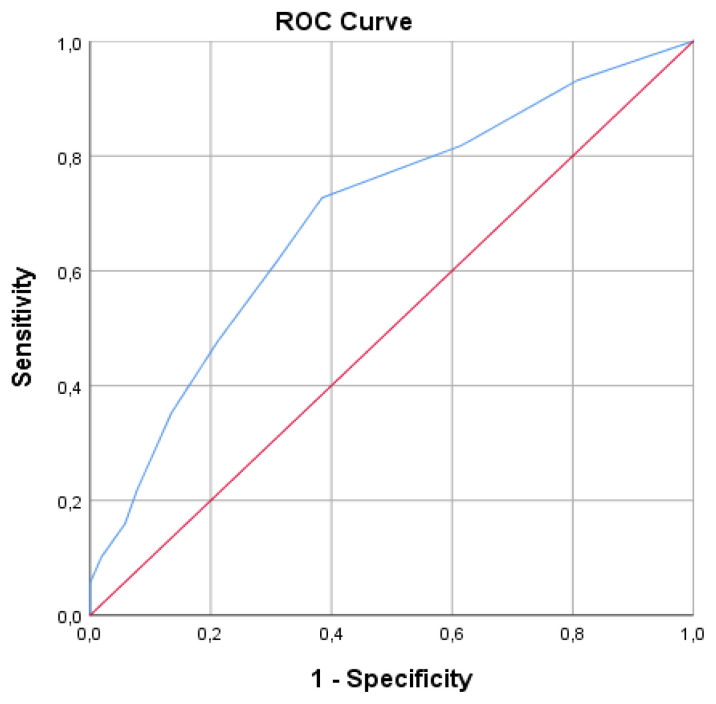
ROC curve assessing the specificity and sensitivity of the skin prick test wheal diameter cut-off point for cat allergy.

The ROC analysis was performed to determine the cut-off value indicating cat allergy in the SPT, and was determined as 5.5 mm with a sensitivity of 72.7% and a specificity of 61.5% (95% CI 60.5%–78.4%) (Figure 2).

Of the 38 patients who had cats at home, 21 (55.26%) tried to take precautions by removing their cats from the house and 7 (18.4%) by increasing the frequency of bathing their cats. Overall, 41 (46.5%) of the 88 patients who reduced contact with cats experienced symptom relief. Removal of the cat from the house was more frequently preferred in patients with shortness of breath and runny nose than those who did not have these symptoms (p = 0.010 and p = 0.011, respectively).

Of the 88 symptomatic patients, 76 (86.4%) preferred allergen-specific immunotherapy. However, due to the unavailability of cat-specific immunotherapy in our country, it could not be administered to any of our patients.

## 4. Discussion

Cat sensitizations and allergies have become common in children with increasing cat ownership. Sensitization to cats was found in 4% of allergic children. However, cat allergy has been demonstrated in 63% of them. In children with allergic symptoms, there is a need for cut-off values for the SPT to distinguish whether the symptoms are related to cat allergy. In our study, the cut-off value for cat allergy was 5.5 mm.

The prevalence of sensitization to animals varies by country, time of exposure, and predisposition to atopy. In Europe, sensitization to cats has been reported to be approximately 26% in adults and 12.1% in the population over 6 years of age in the United States [13–15]. In a study from our country, in which a total of 2822 patients aged 2–18 years were followed up with a diagnosis of AR, cat sensitivity was found in 9.7% of individuals [16]. In previous publications, it has been reported that children living with cats at home develop sensitization to cats more frequently than children without cats at home. In addition, it has been reported that cat allergens can be transferred to shared areas in the community, especially because allergens adhere to the clothes of people living with cats [7,17]. Today, the percentage of homes with pets can be as high as 20%–65% [5]. In our study, the frequency of sensitization to cats was 4%, while 27.1% of our patients had cats at home. Although allergic symptoms are more common in people who keep cats at home, the symptoms in 54 patients occurred with outdoor exposure. Increased sensitization to cat allergen even in cat-free homes suggested that the cases were exposed to cat allergen. An increase in sensitization to cats can be predicted due to the increasing number of stray cats in Türkiye.

As a result of the structural similarity between cat and dog allergens, it is known that cross-reactivity may develop between them and with other mammals [18]. In a study of adults, additional allergen sensitization was evaluated in patients with allergic rhinitis and/or asthma who had at least one cat at home. Sensitization to cats was found to be accompanied most commonly by sensitization to tree pollen, house dust, and dog allergens [19]. In addition to cat sensitization, 85% of our patients had additional aeroallergen sensitization, most commonly pollen allergens. Although we know that cat allergy is frequently associated with other animal allergies, only 10 (7.1%) of our patients had dog allergy. The high frequency of pollen allergy suggests that cat allergens may be carried with pollen and/or the frequency of sensitization to cats may increase in the presence of atopy.

Apart from respiratory and ocular symptoms caused by contact with cats, dermatologic problems and even severe allergic reactions such as anaphylaxis have been reported [20]. The most common symptoms in our patients were related to the respiratory tract. Approximately one fourth of them were on follow-up with a diagnosis of asthma and one third with a diagnosis of allergic rhinitis. No severe allergic reactions were reported, while skin manifestations were less frequent. Animals are the third most common allergen associated with allergic asthma after mites and pollen [5]. In our study, there was no difference in the incidence of asthma and AR between polysensitized and monosensitized patients. Therefore, we consider that cat-sensitized patients should be carefully monitored for respiratory allergic diseases regardless of additional allergen sensitization.

In a study evaluating 120 adults manifesting the symptoms of AR and/or asthma upon contact with cats, the mean SPT wheal size for cat allergen was 5.5 (min–max: 3–15.5) mm and it was reported that SPT showed high sensitivity and specificity [6]. In a different study in which 3068 patients, 16% of whom were pediatric patients, were evaluated, a 7 mm wheal size was determined for the development of allergic symptoms in cats with 80% PPV [21]. In a study evaluating 60 adult patients with AR, it was reported that a cut-off value of >6.5mm for cat allergy could avoid nasal provocation tests, which are often demanding and time-consuming [22]. While food provocation tests are the gold standard diagnostic method for food allergies, provocation tests for inhaled allergens are of limited practical use. Detection of cat sensitization on SPT in patients who report symptoms after contact with cats is an important clue for the diagnosis of allergic diseases. In our study, cat-sensitized symptomatic patients had significantly higher SPT wheal size for cat allergen than asymptomatic patients (7.47 ± 2.83 vs. 5.61 ± 2.36, p < 0.001). It was also found that a wheal size of 5.5 mm and larger may be a warning sign for the presence of allergic symptoms. Therefore, we think that careful questioning of symptoms at this cut-off value and above may be useful for early detection of allergic diseases.

Although removing the pet from the house is the most effective measure to avoid allergens, different methods such as washing the pet frequently, keeping it out of the bedroom, and cleaning the air using HEPA filters have been tried [9,18,23]. Removal of the cat from the home was preferred among our patients who had runny nose and shortness of breath as their primary symptoms. By restricting contact with cats, roughly half of our patients’ symptoms improved. Allergen-specific immunotherapy has often been utilized in symptomatic patient groups. However, there are problems with their supply in our country. Therefore, this treatment modality has not gone beyond being an effective treatment method that could not be used even in eligible patients.

The biggest limitation of our study is the retrospective design.

Increasing cat allergens in households and public areas (homes, schools, daycare centers, and workplaces) will contribute to an increased incidence of cat allergy. Detection of cat allergy is important in the management of treatment. We think that there are not enough studies evaluating the frequency and diagnostic approach of cat allergies in children. The use of a 5.5 mm cut-off point in the SPT, which is easy to access and can be used frequently in practice, may help in this regard. Data on the frequency and interactions of other aeroallergens in cat-sensitized individuals are lacking. This issue needs to be examined in detail in future studies. In addition, people who have been diagnosed with cat allergy will benefit from reducing contact with cats.

## Figures and Tables

**Table 1:** Demographic, clinical, and laboratory findings of patients according to sensitization status.

		Monosensitization to cat	Multisensitive	p	Total
Sex n (%)	Male	13 (9.2)	81 (57.9)	0.579	94 (67.1)
	Female	8 (5.7)	38 (27.2)		46 (32.9)
Age median (IQR§)		12 (6)	12 (6)	0.863	12 (6)
Presence of AR‡, n (%)		8 (5.7)	43 (30.7)	0.863	51 (36.4)
Presence of asthma, n (%)		3 (2.1)	33 (23.6)	0.194	36 (25.7)
SPT† Wheal size for cat allergen (mean ± SD)		7.09 ± 2.36	6.09 ± 2.88	0.534	7.40 ± 2.85
Total IgE (IU/mL) median (IQR§)		97 (151)	108 (105)	0.696	98.5 (109.25)
AEC* (cells/μL) median (IQR§)		125 (193.5)	148 (209)	0.565	133.5 (194.5)

* AEC, Absolute eosinophil count;

‡ AR, Allergic rhinitis;

† SPT, Skin prick test;

§ , IQR

**Table 2:** Evaluation of laboratory findings in patient groups according to their symptoms and status of cat petting at home.

		Symptomatic	Asymptomatic	p	Cat petting at home		p
					Yes	No	
Sensitivity according to the SPT‡ n (%)	Single	15 (10.71)	6 (4.29)	0.378	8 (5.71)	13 (9.29)	0.286
	Multiple	73 (52.14)	46 (32.86)		30 (21.43)	89 (63.57)	
SPT‡ wheal size for cat allergen (mean ± SD)		7.47 ± 2.83	5.61 ± 2.36	<0.001	8.07 ± 2.61	6.30 ± 2.73	<0.001
Total IgE (IU/mL) median (IQR§)		124.5 (153.5)	61.5 (87.5)	<0.001	142 (191)	90.5 (86.25)	<0.001
AEC* (cells/μL) median (IQR§)		207 (34–720)	104 (10–345)	<0.001	342.5 (285.25)	123.5 (124.5)	<0.001

* AEC, Absolute eosinophil count;

‡ SPT, Skin prick test;

§ , IQR
